# Supplementary figures and images for: RAD54 is essential for RAD51-mediated repair of meiotic DSB in Arabidopsis
Source: PLoS Genet. 2021 May 18;17(5):e1008919. doi: 10.1371/journal.pgen.1008919 (PMC8162660; doi:10.1371/journal.pgen.1008919)

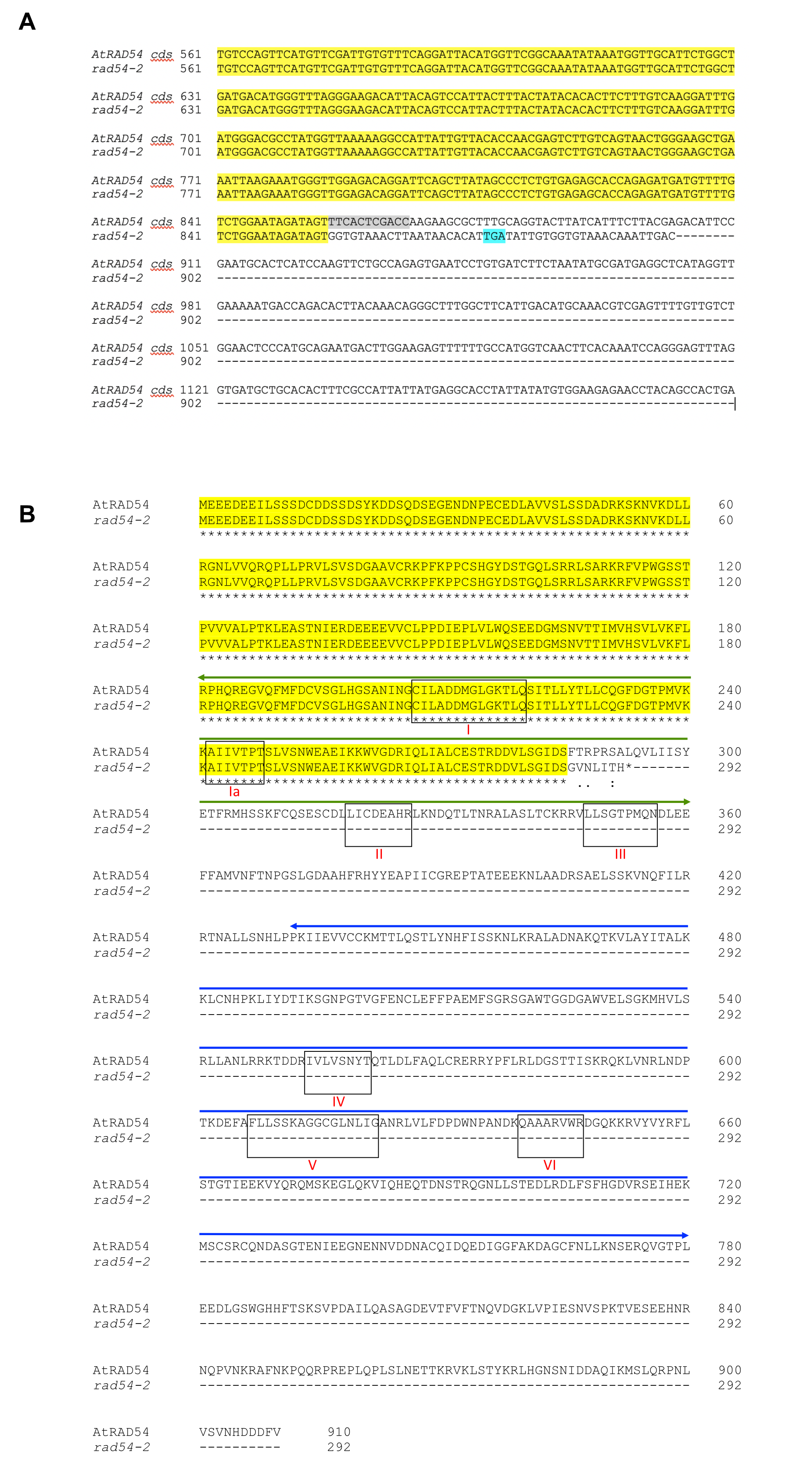

Supplement: S1 Fig — (A) pairwise alignment between AtRAD54 coding sequence (cds) and sequence from T-DNA left border amplification at the T-DNA insertion site in the rad54-2 allele. An in-frame TGA stop codon is highlighted in cyan. T-DNA insertion derived nucleotides deletion is highlighted in grey. (B) Pairwise alignment of the Arabidopsis RAD54 protein and the putative predicted protein from the rad54-2 allele. Alignment was generated using clustalOmega. Numbers indicate amino acid positions. Under the sequences, asterisks, colons and full stops indicate identical, conserved and semi-conserved residues, respectively. The seven conserved ATPase motifs are indicated with black boxes and red roman numerals. These motifs define the two RecA-like domains (parts defined by green and blue lines, respectively), which constitute the “core” translocation motor domains. If translated, the truncated protein from the rad54-2 allele would lack these two recA-like domains. (TIFF) [file pgen.1008919.s001.tiff]

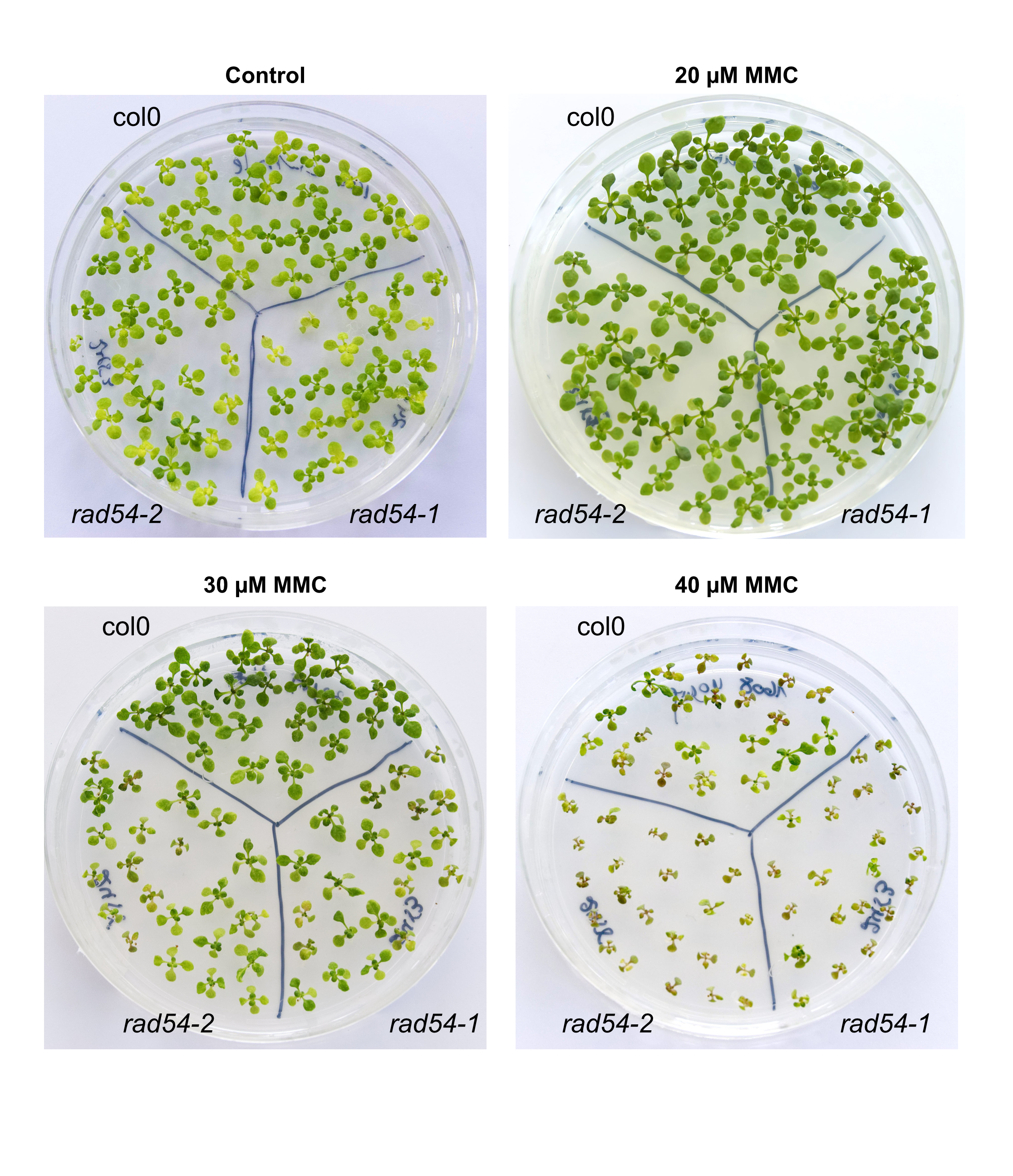

Supplement: S2 Fig — Shown are representative photographs of two-week-old seedlings grown without or with the indicated concentrations of MMC. (TIFF) [file pgen.1008919.s002.tiff]

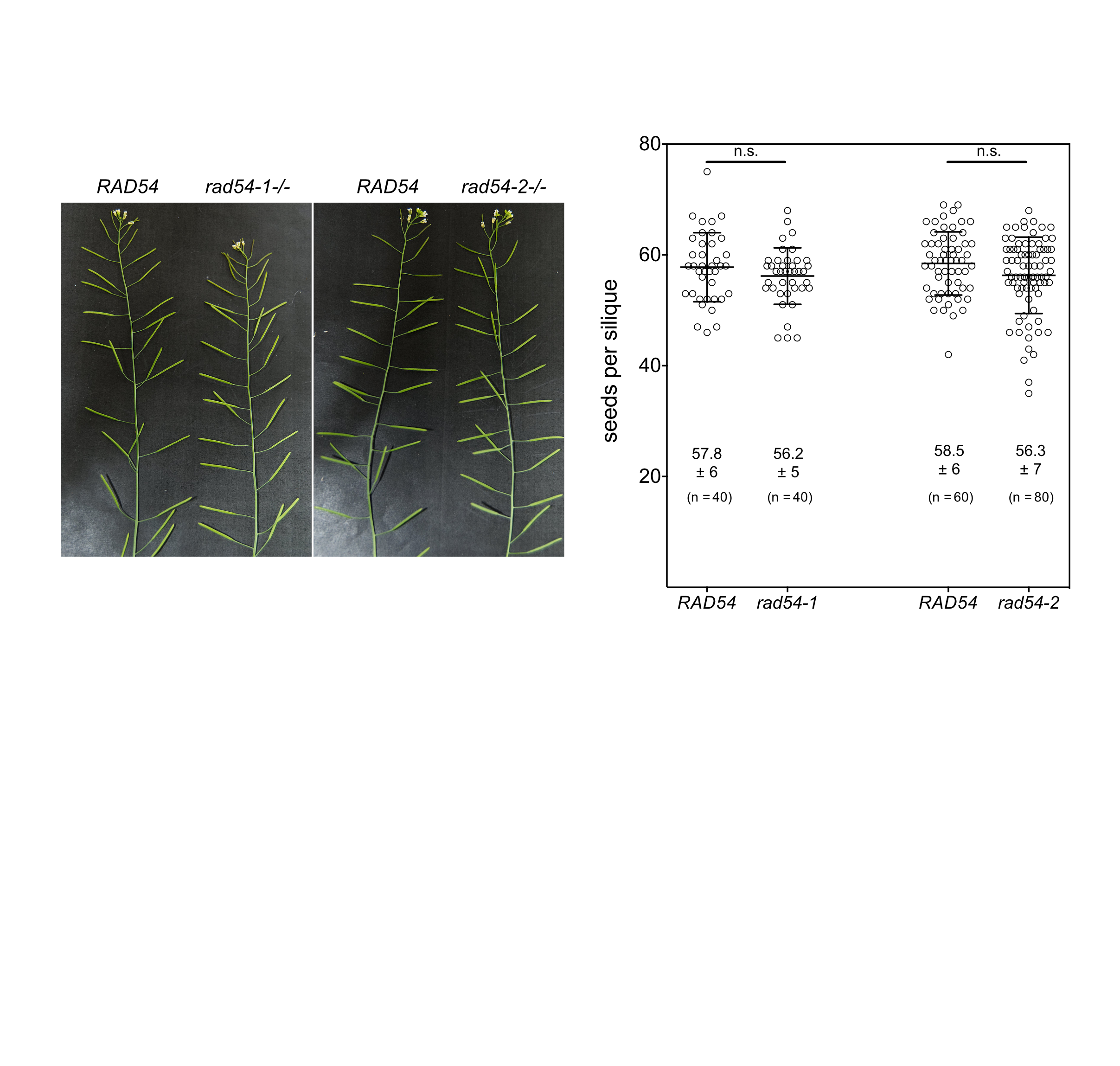

Supplement: S3 Fig — (A) pictures of wild-type and rad54 mutant siliques. (B) Number of seeds per silique in Wild-type, rad54-1 and rad54-2 mutants. Each point represents the number of seeds in one silique. Bars indicate mean ± SD. n.s.: not significantly different. P > 0.05 (unpaired, two-tailed Mann-Whitney test). (TIFF) [file pgen.1008919.s003.tiff]

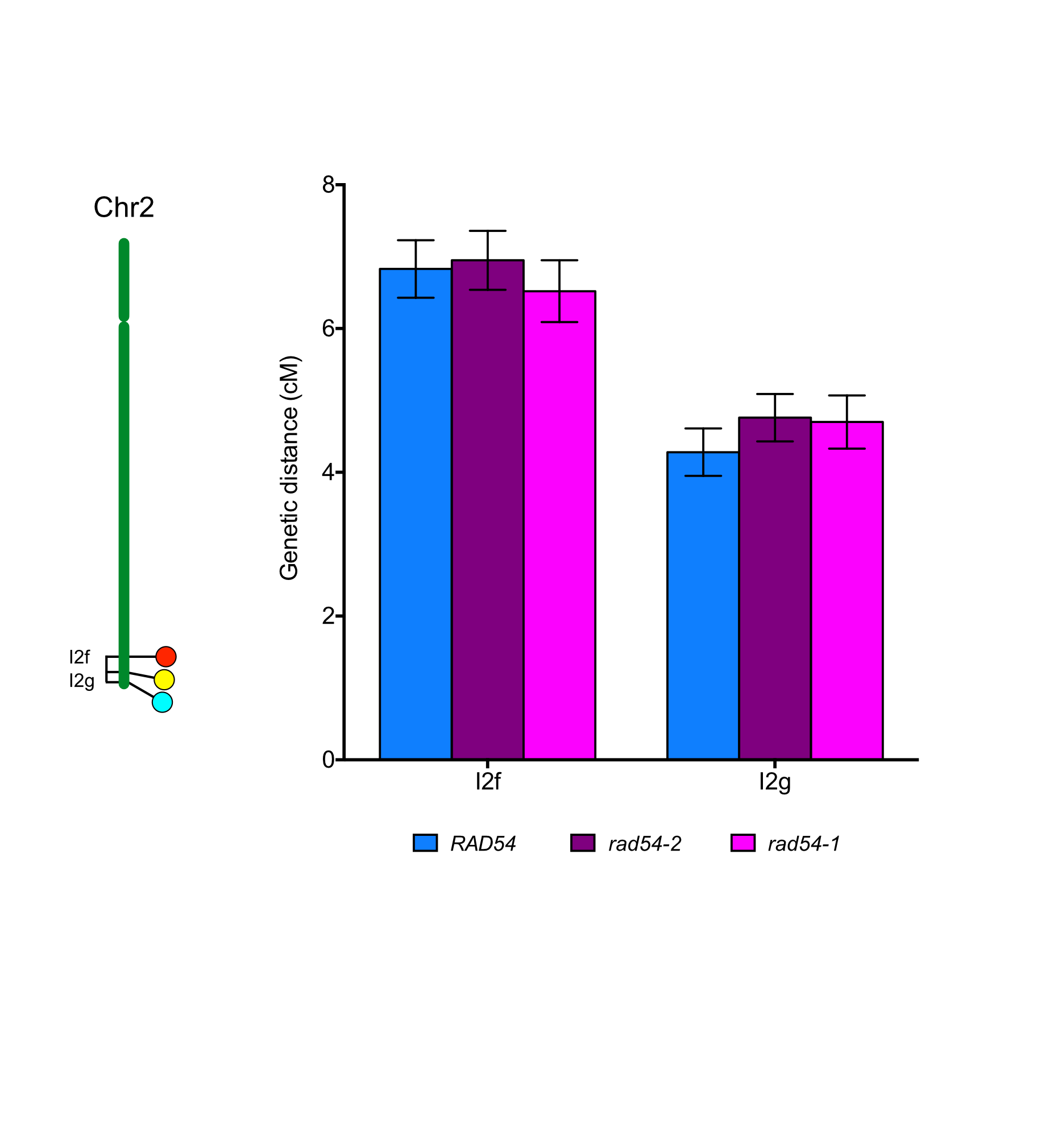

Supplement: S4 Fig — Genetic distances (in centiMorgans, cM) calculated from tetrad analysis of the I2f and I2g intervals on chromosome 2. Bars indicate mean ± SD. For both intervals, WT and rad54 plants do not significantly differ (p<0.05; Z-test). (TIFF) [file pgen.1008919.s004.tiff]

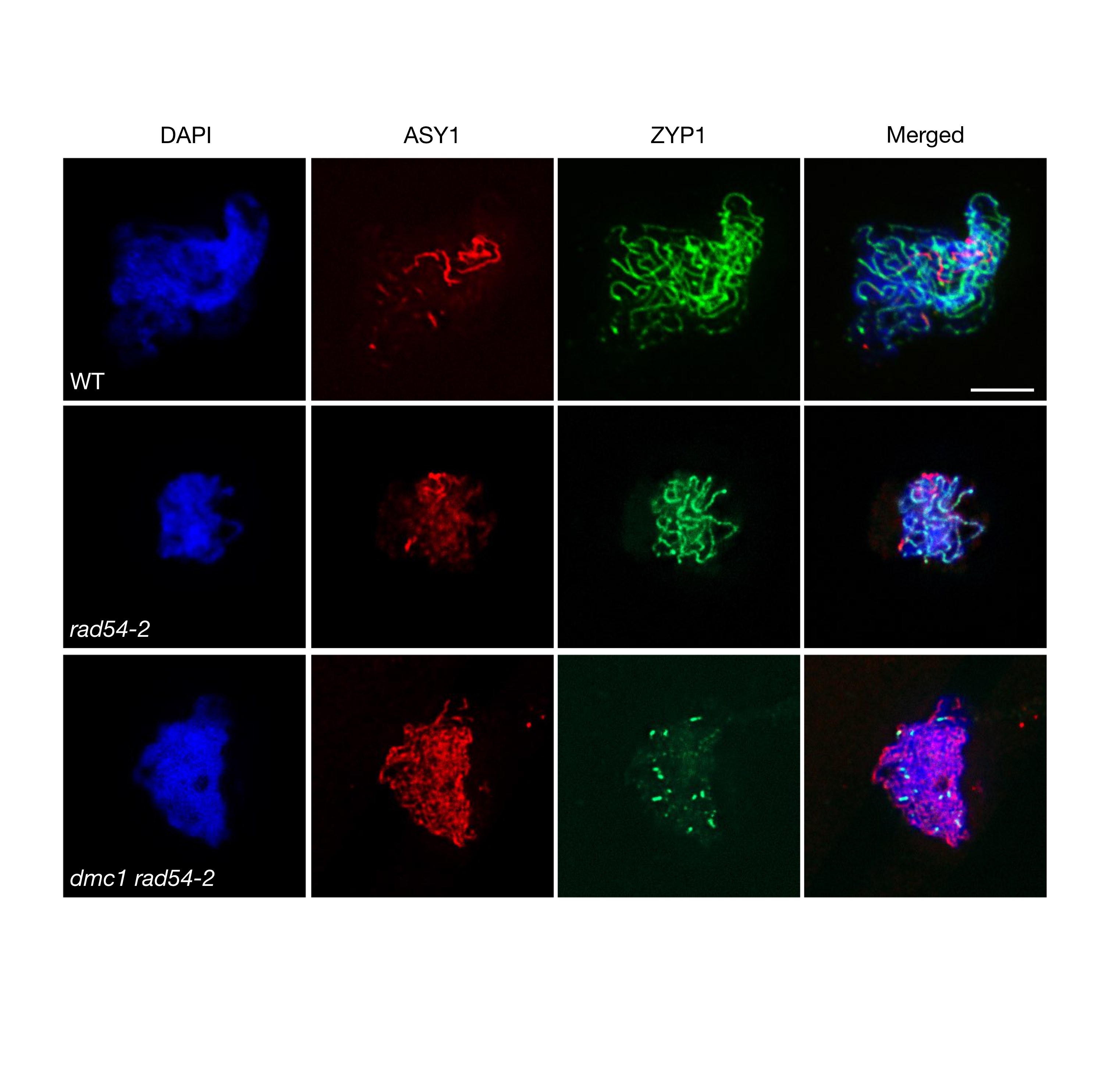

Supplement: S5 Fig — Immunolocalization in wild-type, rad54-2 and dmc1 rad54-2 meiocytes shows that synaptonemal complex transverse filament protein, ZYP1, is not correctly loaded along chromosome axes in dmc1 rad54-2 indicating lack of synapsis. DAPI (blue), ASY1 (red), ZYP1 (green) and merged images are shown. (Scale Bar: 5 μm). (TIFF) [file pgen.1008919.s005.tiff]

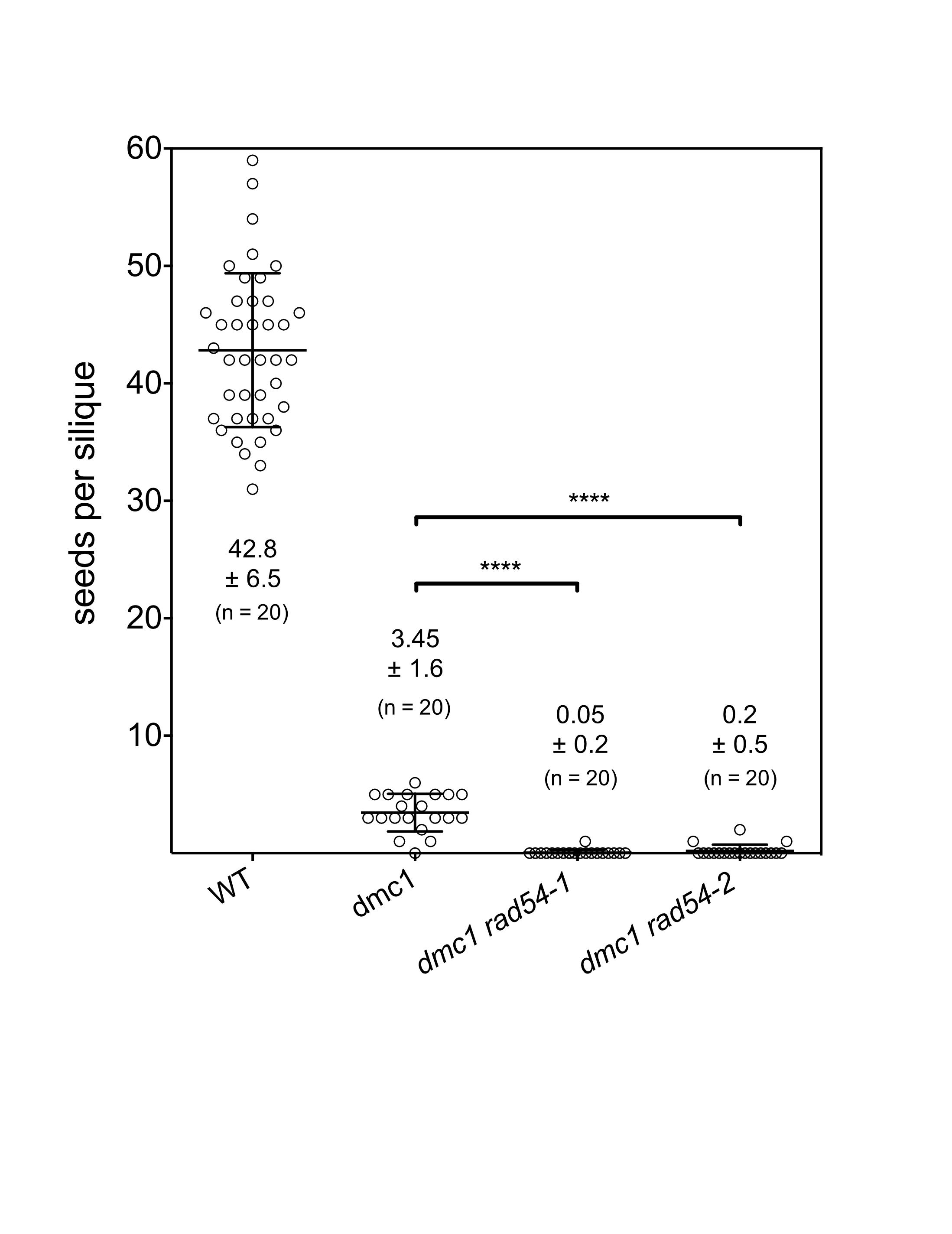

Supplement: S6 Fig — Number of seeds per silique in Wild-type, dmc1, dmc1 rad54-1 and dmc1 rad54-2 mutants. Each spot represents the number of seeds in one silique. Bars indicate mean ± SD. ****: significantly different. P < 0.0001 (unpaired, two-tailed Mann-Whitney test). (TIFF) [file pgen.1008919.s006.tiff]

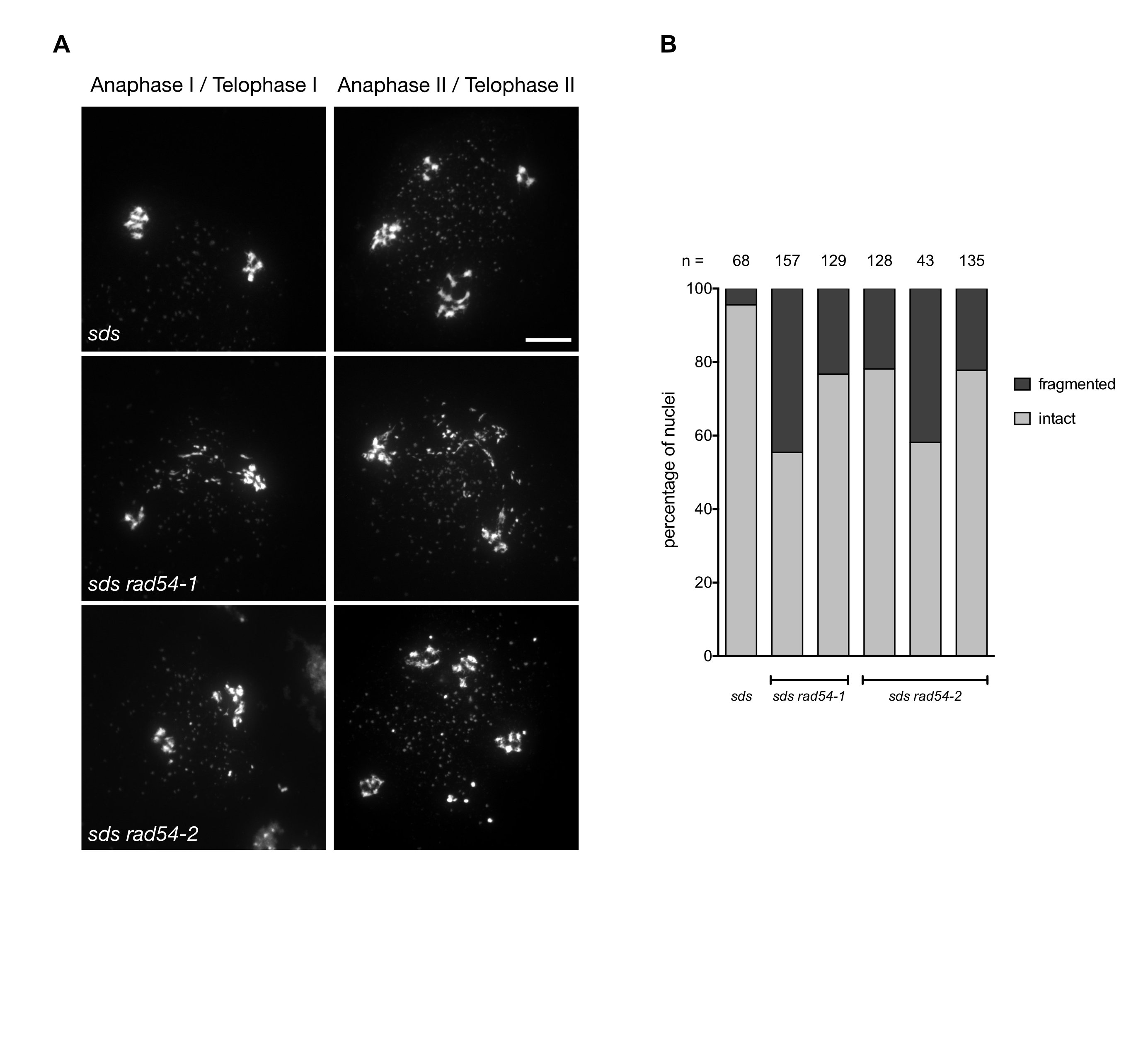

Supplement: S7 Fig — (A) Male meiosis is shown in wild-type, sds rad54-1, and sds rad54-2. Chromosomes were spread and stained with DAPI. (Scale bar = 10 μm). (B) Quantification of male meiocytes showing intact chromosomes or fragmentation. Each bar represents one plant. (TIFF) [file pgen.1008919.s007.tiff]

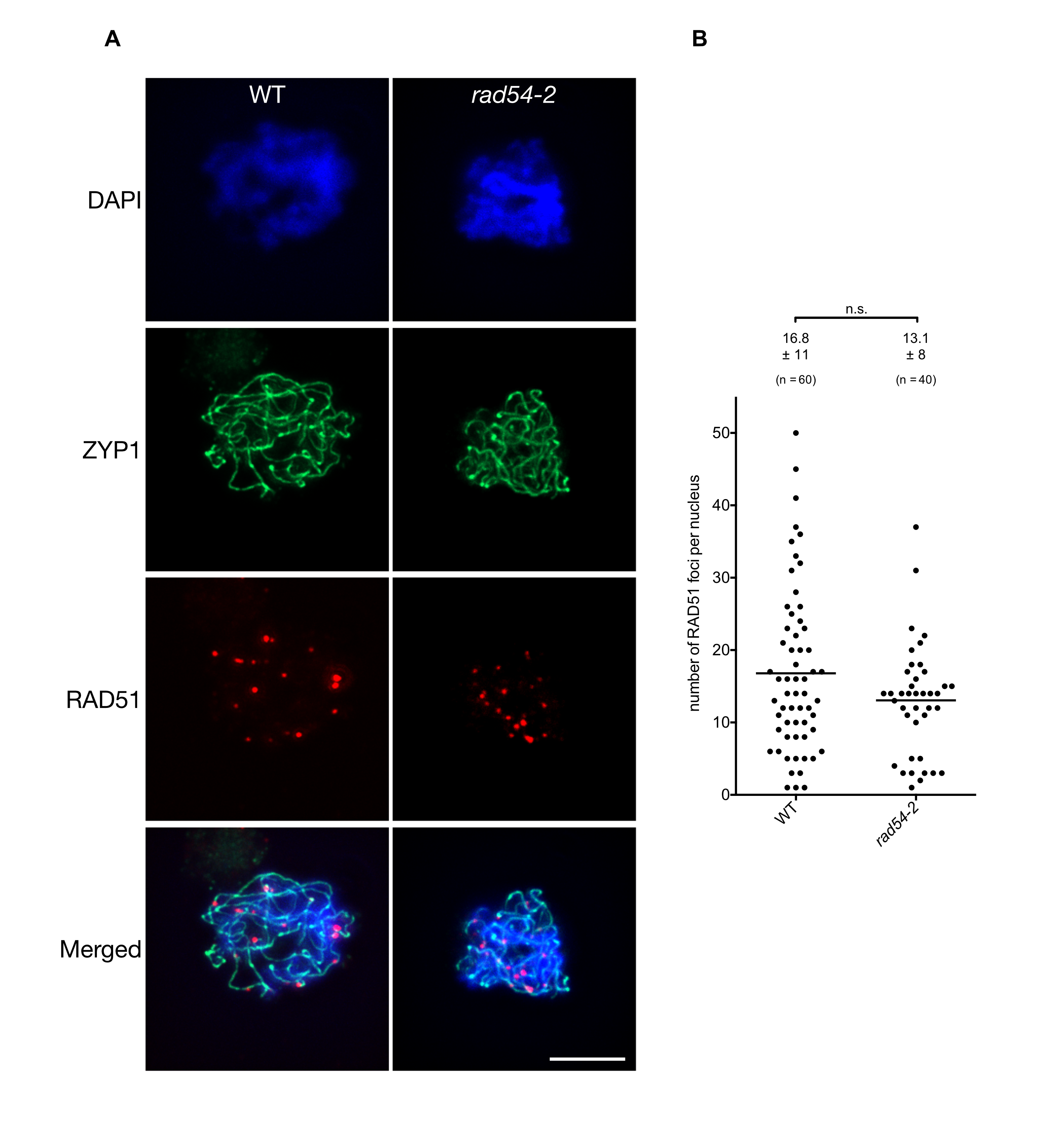

Supplement: S8 Fig — (A) Dual immunolocalization of ZYP1 (green) and RAD51 (red) in wild-type and rad54-2 pachytene cells. (Scale Bar: 5 μm). (B) Quantification of RAD51 foci per positive pachytene cell in wild-type and rad54-2 mutants. Means ± SD are indicated. n.s.: not significantly different (p-value > 0.05, unpaired, two-tailed Mann-Whitney test). (TIFF) [file pgen.1008919.s008.tiff]
